# De Novo *KRAS* G12C–Mutant SCLC: A Case Report

**DOI:** 10.1016/j.jtocrr.2022.100306

**Published:** 2022-03-19

**Authors:** Meridith L. Balbach, Rosana Eisenberg, Wade T. Iams

**Affiliations:** a Division of Hematology-Oncology, Department of Medicine, Vanderbilt University Medical Center, Nashville, Tennessee; b Vanderbilt University School of Medicine, Nashville, Tennessee; c Vanderbilt-Ingram Cancer Center, Nashville, Tennessee; d Department of Pathology, Microbiology, and Immunology, Vanderbilt University Medical Center, Nashville, Tennessee

**Keywords:** KRAS G12C, Small cell lung cancer, Targeted therapy, Case report

## Abstract

The application of KRAS G12C inhibitors in the setting of NSCLC represents a major milestone for a previously “undruggable” target. Here, we present the second reported case of de novo *KRAS* G12C–mutant primary SCLC. Would our patient benefit from a KRAS G12C inhibitor?

## Introduction

The well-known “undruggable” label of Ras has finally been overcome with the recent approval of the KRAS G12C inhibitor sotorasib by the Food and Drug Administration as second-line or later therapy in patients with NSCLC having *KRAS* G12C mutations. With the increasing adoption of tissue-agnostic therapies, the interest in using KRAS G12C inhibitors in additional settings is growing.^1^

Genomic profiles of SCLC revealed rare *KRAS*-activating mutations at a frequency of 0% to 5%, most often G12D and G13D variants.^2^ Whereas the transformation to SCLC has been reported in KRAS G12C–mutant NSCLC, in this brief report, we present the second reported case of de novo *KRAS* G12C–mutant primary SCLC.^3^

## Case Presentation

**Figure 1:**
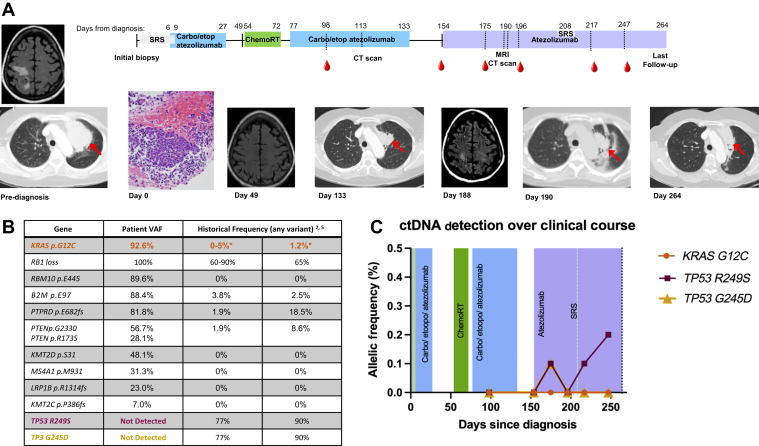
The patient’s clinical course with radiographic and ctDNA monitoring. The patient’s clinical course until the most recent follow-up is illustrated. The colored bars represent active treatment periods (light green = SRS; blue = carboplatin, etoposide, atezolizumab; dark green = concurrent chemoradiation with carboplatin and etoposide; purple = maintenance atezolizumab). The red dots indicate blood collection time points. Direct smear and cell block (illustrated) H&E stain detailing cytologic features typical of small cell carcinoma. (*B*) The molecular genetic testing results with specific alterations detected in initial biopsy specimen are represented. The relative frequency reported in seminal publications by Rudin et al.^2^ (left) and George et al.^4^ (right) of each mutation are shown. (*C*) Percentage mutant allele frequencies of all pathogenic variants detected in ctDNA are shown. Variants of unknown significance identified in ctDNA include RB1 E629D, PTCH1 I374V, and NTRK1 H291P. Colored boxes indicate treatment periods as above. Pathogenic *TP53* variants have been detected only at low allele frequencies, possibly representing CHIP. No KRAS G12C has been detected in ctDNA to-date. Carbo, carboplatin; ChemoRT, chemoradiotherapy; CHIP, clonal hematopoiesis of indeterminate potential; CT, computed tomography; ctDNA, circulating tumor DNA; etop, etoposide; H&E, hematoxylin and eosin; MRI, magnetic resonance imaging; SRS, stereotactic radiosurgery; VAF, variant allele frequency.

A 71-year-old woman with a history of stage I breast cancer 23 years before experienced 2 weeks of decreased left upper extremity coordination followed by a generalized seizure. Brain magnetic resonance image revealed multiple right frontoparietal hemorrhagic metastases (Fig. 1*A*). Subsequent computed tomography of the chest, abdomen, and pelvis revealed an 11-cm solid mass in the left upper lobe (LUL). Fine needle aspiration of the LUL mass was performed and separately reviewed by the MD Anderson Cancer Center, Memorial-Sloan Kettering Cancer Center and Vanderbilt University Medical Center, which revealed SCLC (Fig. 1*A*). No distant metastases were noted on positron emission tomography scan. The patient was training for a triathlon, with an Eastern Cooperative Oncology Group performance status of 0 on diagnosis. She quit smoking 41 years before diagnosis with a 10 pack-year smoking history.

On the basis of the unusual presentation, Tempus xT molecular genetic testing (Tempus, Chicago, Illinois) was performed, assessing 648 cancer-related genes in an initial tumor sample with matched normal tissue.^5^ This revealed a gain of function G12C missense variant in *KRAS* with a very high variant allele frequency (VAF) (92.6%). Copy number loss of *RB1* and somatic loss of function variants were also noted, as detailed in Figure 1*B* were also noted.

Urgent stereotactic radiosurgery to the brain metastases was completed. The patient was started on carboplatin/etoposide/atezolizumab and underwent consolidative thoracic radiation*.* After five cycles of systemic therapy, response imaging revealed an interval decrease in the LUL mass from 5.2 by 7.6 cm to 4.0 by 3.4 cm, signifying a partial response by Response Evaluation Criteria in Solid Tumors criteria. Subsequent Tempus xF (Tempus) circulating tumor DNA (ctDNA) analysis of 105 cancer-related genes in peripheral blood samples did not reveal pathogenic alterations present in the original tumor specimen, likely because of treatment-induced ctDNA clearance (Fig. 1*C*).

Given her favorable response to initial treatment, the patient received maintenance atezolizumab. The most recent positron emission tomography imaging revealed a continued decrease in size and avidity of the primary tumor. However, during maintenance atezolizumab treatment, the patient developed a concern for leptomeningeal enhancement on brain magnetic resonance imaging. Lumbar puncture cytology was negative for malignant cells, and the patient completed stereotactic radiosurgery to the small site of concern.

Although the patient remains clinically stable on standard extensive-stage SCLC therapy, she is being considered for a KRAS G12C inhibitor clinical trial (NCT04185883) at progression, given the high VAF in the initial tissue sample.

## Discussion

Canonically, loss of function mutations in *TP53* and *RB1* have been regarded as nearly ubiquitous in SCLC.^4^ In contrast, activating *KRAS* mutations are rare in SCLC, with a historical frequency of 0% to 5%. Interestingly, our patient lacked a *TP53* mutation in the diagnostic specimen (though *RB1* loss was observed). The appearance of a *TP53* mutation at low VAF during therapy raises the question of clonal hematopoiesis of indeterminate potential or SCLC subtype plasticity during therapy.^6^ With an improving understanding of SCLC subtypes, it has become apparent that a subgroup of SCLC tumors exhibits a more “non-neuroendocrine” phenotype.^6^ These non-neuroendocrine SCLC subtype tumors typically express POU2F3 or YAP1 and seem more peripherally-located in the pulmonary parenchyma compared with classic, centrally located, neuroendocrine-type SCLC (neuroendocrine SCLC is often ASCL1-positive rather than POU2F3- or YAP1-positive). We would hypothesize that this patient’s SCLC began as a predominantly non-neuroendocrine subtype, likely harboring a closer relationship to a shared cell of origin (type II alveolar cell), with a typical pulmonary adenocarcinoma and susceptible to *KRAS* driver mutations.^7^

Our patient with KRAS G12C-mutant SCLC has responded well to standard treatment, now with a progression-free survival of 8.6 months compared with the previously reported median progression-free survival of 5.2 months in this setting, which is congruent with a more non-neuroendocrine behavior.^8^ The successful application of KRAS G12C–targeted therapy in NSCLC presents an exciting opportunity to achieve better outcomes in patients with *KRAS* G12C mutations and other histologic types. Unfortunately, there is no data to guide on the use of KRAS G12C inhibitors in patients with SCLC.

The National Comprehensive Cancer Network guidelines recommend routine molecular testing for actionable biomarkers before administering first-line therapy for all patients with NSCLC. In contrast, current National Comprehensive Cancer Network guidelines for SCLC do not recommend molecular testing at any point in a patient’s disease course. The discovery of a noncanonical, yet potentially actionable, genetic alteration in our patient with SCLC may argue for expanded molecular testing across tumor types as the number of available targeted therapies rapidly increases.

## Conclusions

Here, we present the second reported case of a de novo *KRAS* G12C mutation in histologically-proven primary SCLC. Whereas the patient is currently exhibiting a good response to first-line therapy, she will present a unique challenge at first progression: would she benefit from KRAS G12C inhibitor therapy? Unlike NSCLC, SCLC has no approved targeted therapies by the Food and Drug Administration and observations of very rare mutations targetable in other histologic types raise the recurring question of therapeutic vulnerability with the lack of even small clinical trial data.

## CRediT Authorship Contribution Statement

**Meridith L. Balbach:** Conceptualization, Writing - original draft, Visualization, Project administration.

**Rosanna Eisenberg:** Resources, Investigation.

**Wade T. Iams:** Conceptualization, Resources, Writing - review & editing, Supervision.

## References

[1] Hong D.S., Fakih M.G., Strickler J.H. (2020). KRASG12C inhibition with Sotorasib in advanced solid tumors. N Engl J Med.

[2] Rudin C., Durinck S., Stawiski E. (2012). Comprehensive genomic analysis identifies SOX2 as a frequently amplified gene in small-cell lung cancer. Nat Genet.

[3] Kodaz H., Taştekin E., Erdoğan B. (2016). KRAS mutation in small cell lung carcinoma and extrapulmonary small cell cancer. Balk Med J.

[4] George J., Lim J., Jang S. (2015). Comprehensive genomic profiles of small cell lung cancer. Nature.

[5] Beaubier N., Tell R., Lau D. (2019). Clinical validation of the tempus xT next-generation targeted oncology sequencing assay. Oncotarget.

[6] Rudin C.M., Poirier J.T., Byers L.A. (2019). Molecular subtypes of small cell lung cancer: a synthesis of human and mouse model data. Nat Rev Cancer.

[7] Sutherland K.D., Proost N., Brouns I., Adriaensen D., Song J.Y., Berns A. (2011). Cell of origin of small cell lung cancer: inactivation of Trp53 and Rb1 in distinct cell types of adult mouse lung. Cancer Cell.

[8] Horn L., Mansfield A.S., Szczęsna A. (2018). First-line atezolizumab plus chemotherapy in extensive-stage small-cell lung cancer. N Engl J Med.

